# Neural Correlates of Racial Ingroup Bias in Observing Computer-Animated Social Encounters

**DOI:** 10.3389/fnhum.2017.00632

**Published:** 2018-01-04

**Authors:** Yuta Katsumi, Sanda Dolcos

**Affiliations:** ^1^Department of Psychology, University of Illinois at Urbana–Champaign, Champaign, IL, United States; ^2^Beckman Institute for Advanced Science and Technology, University of Illinois at Urbana–Champaign, Urbana, IL, United States

**Keywords:** first impression, non-verbal behavior, race, group membership, fMRI

## Abstract

Despite evidence for the role of group membership in the neural correlates of social cognition, the mechanisms associated with processing non-verbal behaviors displayed by racially ingroup vs. outgroup members remain unclear. Here, 20 Caucasian participants underwent fMRI recording while observing social encounters with ingroup and outgroup characters displaying dynamic and static non-verbal behaviors. Dynamic behaviors included approach and avoidance behaviors, preceded or not by a handshake; both dynamic and static behaviors were followed by participants’ ratings. Behaviorally, participants showed bias toward their ingroup members, demonstrated by faster/slower reaction times for evaluating ingroup static/approach behaviors, respectively. At the neural level, despite overall similar responses in the action observation network to ingroup and outgroup encounters, the medial prefrontal cortex showed dissociable activation, possibly reflecting spontaneous processing of ingroup static behaviors and positive evaluations of ingroup approach behaviors. The anterior cingulate and superior frontal cortices also showed sensitivity to race, reflected in coordinated and reduced activation for observing ingroup static behaviors. Finally, the posterior superior temporal sulcus showed uniquely increased activity to observing ingroup handshakes. These findings shed light on the mechanisms of racial ingroup bias in observing social encounters, and have implications for understanding factors related to successful interactions with individuals from diverse backgrounds.

## Introduction

Dramatic changes in the racial and ethnic composition of the United States have made social interactions with diverse social groups ubiquitous elements of everyday life (Cárdenas et al., 2011). An essential component of these interactions, with a critical influence on drawing inferences about others, is represented by non-verbal behaviors (Murphy, 2012), the perception of which can be significantly influenced by whether or not one shares racial/ethnic identities with the others (*in*group vs. *out*group, respectively) (Elfenbein and Ambady, 2002; Adams et al., 2010). For instance, non-verbal information from ingroup members tends to be decoded more accurately than that from outgroup members (Elfenbein and Ambady, 2002; Adams et al., 2010), possibly reflecting greater familiarity and less uncertainty associated with ingroup non-verbal behaviors (Dovidio and Gluszek, 2012). Extending prior behavioral evidence regarding the impact of group membership on social cognition, recent functional neuroimaging studies have identified a network of brain regions showing sensitivity to ingroup vs. outgroup differences in various task contexts (Molenberghs, 2013; Shkurko, 2013). However, neural mechanisms associated with observing different kinds of non-verbal behaviors displayed by ingroup vs. outgroup members in a defined social context remain relatively unclear. The present study addressed this important issue by using a novel experimental paradigm (Sung et al., 2011; Dolcos et al., 2012; Katsumi et al., 2017), in which participants observed and evaluated a series of non-verbal social encounters with racially ingroup and outgroup characters in a business context.

Recent neuroimaging studies have identified dissociable engagement of a network of regions associated with processing information about ingroup vs. outgroup members (Kubota et al., 2012; Molenberghs, 2013; Shkurko, 2013; Amodio, 2014). These modulations of neural responses by group membership have been observed in various task contexts (e.g., social categorization, face/action perception, empathy), and are thought to reflect either relatively automatic/bottom-up or deliberate/top-down processes (Molenberghs, 2013). Several brain regions have been identified as showing differential activation linked to processing information about ingroup and outgroup members. On the one hand, rapid detection and categorization of group membership may be subserved by regions such as the amygdala and fusiform gyrus, particularly when a task involves categorization of face-based stimuli (Cunningham et al., 2004; Van Bavel et al., 2008). There is evidence suggesting that the amygdala shows greater activation to either ingroup or outgroup stimuli, depending on the motivational significance associated with different goals to process them (Cunningham and Brosch, 2012; Amodio, 2014). In contrast, the fusiform gyrus seems to be preferentially activated in response to ingroup faces (Van Bavel et al., 2008).

On the other hand, processing of ingroup vs. outgroup members may also involve more deliberate monitoring and regulation of prepotent biases (e.g., negative associations with outgroup members), and these processes have been linked to increased activity in the anterior cingulate cortex (ACC) and lateral frontal regions [e.g., dorsolateral prefrontal cortex (dlPFC)], respectively (Kubota et al., 2012; Amodio, 2014). The ACC and dlPFC share numerous functional connections, and together they are involved in various aspects of top-down control and decision-making processes (Amodio and Frith, 2006). Taken together, these findings suggest that processing of ingroup and outgroup members is linked to modulation of activity in a network of regions implicated in various social cognitive and emotional processes, both at the level of relatively automatic/bottom-up and deliberate/top-down processing.

Consistent with the notion that processing of outgroup members tends to involve more conscious regulatory mechanisms, previous research suggests that processing of ingroup members tends to occur relatively more spontaneously than that of outgroup members (Bernstein et al., 2007; Van Bavel et al., 2008; Senholzi and Ito, 2013). For instance, categorization of ingroup faces is associated with faster reaction time (RT) than that of outgroup faces (Van Bavel et al., 2008; Ratner and Amodio, 2013), and electrophysiological evidence also demonstrates enhanced early perceptual processing of ingroup relative to outgroup faces when race is a salient feature (Ratner and Amodio, 2013; Senholzi and Ito, 2013). Such patterns of ingroup bias may be due to additional psychological significance afforded by ingroup membership, which contributes to one’s social identity and self-esteem (Tajfel and Turner, 1986; Brewer, 1999).

Not surprisingly, neural ingroup bias has been identified in fMRI studies in which greater activation in regions implicated in socioemotional processes was observed for ingroup than outgroup stimuli in the context of social categorization (Van Bavel et al., 2008; Morrison et al., 2012; Molenberghs and Morrison, 2014), non-verbal perception (Freeman et al., 2009; Adams et al., 2010), and impression formation (Freeman et al., 2010). In addition to the fusiform gyrus discussed above, greater response in regions such as the medial prefrontal cortex (mPFC), superior temporal sulcus (STS), temporo-parietal junction (TPJ), and insula has been identified as neural signatures of ingroup favoritism (reviewed in Molenberghs, 2013). Importantly, the degree of activation in these regions has been associated with behavioral measures indicating preference for ingroup members, thus lending support to the idea that activity in these regions provides a unique index of ingroup bias in social cognition and behavior (Van Bavel et al., 2008; Molenberghs et al., 2013). Taken together, extant evidence suggests that enhanced processing of ingroup members occurs possibly because of increased psychological and social significance associated with ingroup identification. Neural ingroup bias, as typically reflected in greater activation for processing ingroup than outgroup stimuli, does not appear to be confined to a single region, but rather seems to manifest as modulations of functional networks broadly implicated in social cognitive and emotional processes (Molenberghs, 2013).

As summarized above, previous studies have documented the neural correlates of ingroup bias in various task contexts. However, several issues remain unclear regarding the role of racial group membership in the neural correlates of non-verbal perception and impression formation. First, our current understanding of the neural correlates of processing group membership is largely based on evidence from studies using static pictures depicting faces in isolation, particularly in the context of social categorization (e.g., Shkurko, 2013). In real-life situations, however, inference of others’ mental states is usually based on more comprehensive evaluations of non-verbal behaviors through both facial and bodily expressions (Dolcos et al., 2012; Van den Stock et al., 2015b; Katsumi et al., 2017). Second, although a few previous studies have identified dissociable neural responses associated with observing dynamic gestures displayed by racial ingroup vs. outgroup members (e.g., Gutsell and Inzlicht, 2010; Losin et al., 2012), these studies often lacked a well-defined social context in which these cues were embedded and processed. Importantly, categorization of ingroup vs. outgroup members is often fluid and context-dependent (Turner et al., 1994), and there is in fact evidence showing that dissociable neural sensitivity to ingroup vs. outgroup members can be modulated by task goals (e.g., superficial categorization vs. individuated processing) (Wheeler and Fiske, 2005; Freeman et al., 2010). Therefore, clarification of these issues is of particular importance in better understanding the neural correlates of processing racial ingroup vs. outgroup information in a defined social context with increased ecological validity.

The main goal of the present investigation was to clarify the neural mechanisms associated with observing social encounters with racially ingroup and outgroup characters displaying different types of whole-body non-verbal behaviors. Specifically, we incorporated both *dynamic* and *static* displays of non-verbal behaviors, and placed them in a socially relevant (i.e., business) context. Dynamic social interactions between the characters consisted of approach and avoidance behaviors that encouraged or discouraged further interaction, respectively, which were preceded or not by a handshake, a customary greeting behavior in business settings in North America. Approach and avoidance behaviors were included given prior evidence linking processing of ingroup vs. outgroup members to approach vs. avoidance tendencies, where ingroup favoritism may manifest as greater associations between ingroup and approach/positive behaviors (Paladino and Castelli, 2008). Handshakes were also included as a common greeting behavior signaling approaching intentions in Western cultures (Dolcos et al., 2012; Katsumi et al., 2017). Moreover, static social scenes involved observation of characters depicted on a cardboard cutout, thus mimicking real-life situations in which the human presence is replaced by similar cardboard images (e.g., of popular people or an organization’s employees), such as those posted in stores or banks.

Based on the extant evidence, we tested the following hypotheses. Regarding the behavioral effects, (1) we expected to observe differences in behavioral responses (i.e., ratings and/or RTs) reflecting bias toward ingroup members, as observed in previous studies of ingroup processing (Bernstein et al., 2007; Van Bavel et al., 2008; Molenberghs et al., 2013). Regarding the fMRI results, (2) we expected that observing ingroup and outgroup dynamic social interactions would engage a broader network of regions involved in action observation and social cognition, including the posterior STS (pSTS), extrastriate body area (EBA), fusiform gyrus, as well as lateral and medial frontal regions (e.g., inferior frontal gyrus) (Dolcos et al., 2012; Van den Stock et al., 2015b; Yang et al., 2015). Second, we expected that observing ingroup vs. outgroup social encounters would be associated with dissociable activations in brain regions previously identified in studies of group membership in the context of social categorization, non-verbal perception, or impression formation. More specifically, (3) greater activation to observing social encounters with ingroup than outgroup members was expected in regions previously identified as showing sensitivity to ingroup information, such as the fusiform gyrus, mPFC, and pSTS/TPJ (Van Bavel et al., 2008; Adams et al., 2010; Molenberghs, 2013). In particular, we expected modulation of activity in these regions associated with observing ingroup approach behaviors and handshakes (Paladino and Castelli, 2008; Freeman et al., 2009). Finally, (4) increased activity for observing outgroup vs. ingroup members was also expected in regions typically involved in cognitive control and regulatory processes, including the ACC and dlPFC (Kubota et al., 2012), possibly linked to observing avoidance behaviors.

## Materials and Methods

### Participants

Twenty right-handed healthy young adults (10 women; age 18–29) participated in the study. All participants were native English speakers, identified their race as Caucasian/White, and had no history of neurological, psychological, or psychiatric disorders. The experimental protocol was approved by the University of Illinois Institutional Review Board, and all participants provided written informed consent and received payment for their participation.

### Experimental Design

Stimuli consisted of movies similar to those used in a previous investigation from our group (Dolcos et al., 2012), supplemented by additional movies incorporating clear manipulations of characters’ race (see also Katsumi et al., 2017). Stimuli were created in Poser 7.0^1^, and presented using the CIGAL software (Voyvodic, 1999). The Poser software package has been used in a number of previous studies examining the neural correlates of social interaction with virtual characters (Pelphrey et al., 2004; Mojzisch et al., 2006; Schilbach et al., 2006; Muhlberger et al., 2009; Pitskel et al., 2011; Zucker et al., 2011). Through manipulations of various non-verbal behaviors displayed by animated characters, these studies identified modulation of activity in brain regions including the mPFC and STS (e.g., Pelphrey et al., 2004; Schilbach et al., 2006). Importantly, the role of these regions has been similarly identified in neuroimaging studies of social interactions with real humans (Iacoboni et al., 2004; Kujala et al., 2012; Van den Stock et al., 2015b). It is also noteworthy that Poser has been used to manipulate the racial characteristics of virtual characters in the context of social evaluations (Stepanova and Strube, 2009). Collectively, these findings point to the validity of Poser to examine the neural correlates of racial ingroup bias in observing social encounters in the present study.

**FIGURE 1:**
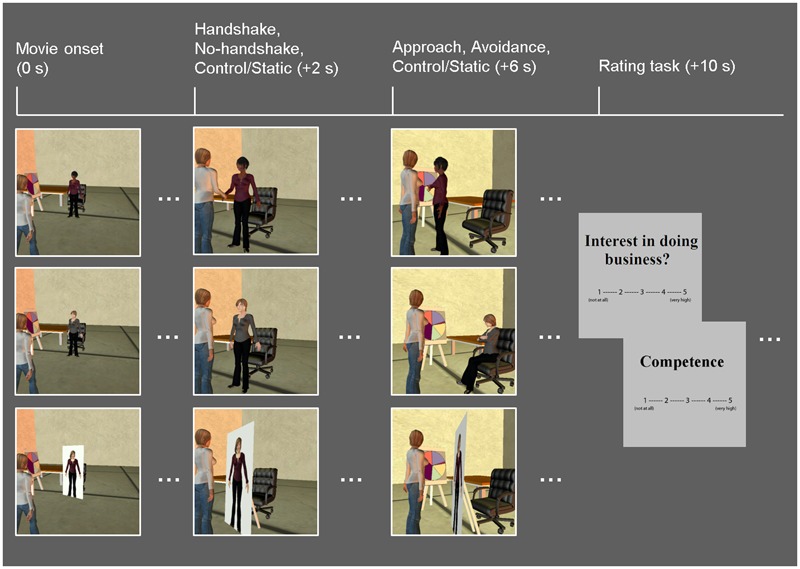
Diagram of the task. Event-related fMRI data were recorded while participants viewed movies of guest–host interactions, in which hosts displayed dynamic non-verbal behaviors that either encourage (approach: open postures, smiley faces; top row) or discourage (avoidance: closed postures, frowny faces; middle row) further social interaction. A static social scene condition, in which the host characters were replaced with cardboard cutouts depicting their whole body, was used as a control condition (bottom row). Half of the dynamic social interaction trials involved a handshake; both the dynamic and static conditions were followed by participants’ ratings of the hosts on competence as business representatives, and their own interest in doing business with the hosts. Time (in seconds) denoted in parentheses above specifies the onset of each event relative to that of each movie.

Similar to the Dolcos et al. (2012) study, the task consisted of a series of 10-s whole-body animated movies illustrating non-verbal guest–host interactions in a business setting (**Figure 1**). Participants viewed the guest being greeted by a host (*dynamic* social interaction condition) or a cardboard cut-out of a host (*static* social scene condition). In the dynamic condition, the host displayed non-verbal behaviors that either encouraged (*approach* condition) or discouraged (*avoidance* condition) further social interactions. Specifically, the host in the Approach condition stepped toward the guest while displaying open postures and smiley faces, whereas the host in the Avoidance condition stepped away from the guest while displaying closed postures and frowny faces. Within each condition, in half of the trials, dynamic social interaction was preceded by a handshake initiated by the host as part of the greeting protocol, and the order of trials with and without a handshake was counterbalanced across participants. In the static social scene condition, the host was depicted on a cardboard cutout, thus mimicking real-life contexts in which the human presence is replaced by similar cardboard images (e.g., of popular people or an organization’s employees), such as those posted in stores or banks. It should be noted that, as in the previous study using similar experimental stimuli (Dolcos et al., 2012), there were no overall differences in the objective motion between the dynamic and static condition movies, nor within the dynamic (approach vs. avoidance) conditions. This was due to the fact that movies in the static condition involved increased panning, which seemingly contributed to changes in luminance as much as biological motion observed in the dynamic conditions.

Host race was manipulated following previous studies using similar procedures (Stepanova and Strube, 2009; Krämer et al., 2013), by applying unique facial characteristics and skin tones representing particular racial groups. Ingroup hosts represented Caucasian individuals, whereas outgroup hosts represented three non-Caucasian racial/ethnic groups: East Asian, South Asian, and African–American, in proportions similar to the representation of these racial/ethnic groups in the local student population (i.e., 50% Caucasian, 18.75% East Asian, 18.75% South Asian, 12.5% African–American). Host race was validated by a subset of the present sample (*n* = 18) as well as an independent sample (*N* = 97), who rated the host’s race in each movie using 10-point scales (1 = *Definitely not Caucasian*, 10 = *Definitely Caucasian*). These participants provided their ratings of host race after they had completed the main evaluation task, in order to avoid task contamination. Results based on the present sample showed that ingroup/Caucasian stimuli were significantly more likely to be perceived as Caucasian (*M* = 8.51, *SD* = 1.13) compared to outgroup/non-Caucasian stimuli (*M* = 3.10, *SD* = 1.25) (*p* < 0.001; similar results were obtained in the other sample), thus confirming our successful manipulation of host race. Ingroup and outgroup hosts consisted of equal proportions of female and male characters. Each movie was followed by rating screens, which prompted participants to provide the following ratings, using 5-point scales (1 = *Not at all*, 5 = *Very high*): business competence of the host (“Competence”) and their own interest in doing business with the host (“Interest in doing business”). Each rating screen was displayed for 2 s, and the order of the ratings was counterbalanced across trials.

### Procedure

As part of the pre-scanning instructions, participants were told that the study examined the effect of first impressions formed in brief social encounters on the subsequent decision to further engage in business relations. No further explanation regarding the reason for choosing this particular context for the task was provided to the participants. Prior to the beginning of the task, participants were instructed to use the whole rating scale and to give their ratings based solely on the observed social encounters, as well as to make their responses as quickly and accurately as possible. In the static social scene condition, participants were told to evaluate the host on a cardboard cutout as if s/he was an agent representing his or her company.

Participants completed eight runs of 20 trials each for a total of 160 trials, and were assigned different run orders. The trials within each run were pseudo-randomized so that no more than three trials of the same kind were presented consecutively. Each run started with 6 s of a fixation screen to allow stabilization of the fMRI signal, and an inter-trial interval of 8 s followed each trial and ended each run. All stimuli appeared against a black background and were projected on a screen directly behind the participant’s head within the scanner, which participants viewed through a mirror. Responses were recorded using a five-button response box placed under the participant’s right hand. Following the scanning session, participants viewed the same set of stimuli again for the validation of host race, after which they were thoroughly debriefed about the true purpose of the study.

### Behavioral Data Analysis

Similar to previous investigations (Van den Stock et al., 2015a), analyses of the rating and RT data were preceded by normality check using Shapiro–Wilk tests. Results confirmed that the frequency distribution of the behavioral data did not significantly differ from a normal distribution (*p*’s > 0.05). Therefore, the use of parametric tests was justified for statistical analyses of the behavioral data. The main goal of behavioral data analyses was to clarify the role of racial group membership in the evaluation of social encounters, with a focus on the effect of observing dynamic (approach/avoidance) social interactions vs. static social scenes and handshake. To this end, we conducted a series of repeated-measures analysis of variance (ANOVAs) to assess the differences in participants’ ratings and RTs using the following manipulations as the independent variables: *Behavior* (approach, avoidance, static), *Handshake* (handshake, no-handshake), and *Host Race* (ingroup, outgroup). The dependent variable in each ANOVA was the average of competence and interest ratings, except for the analyses of RTs where we identified significant effects only related to the interest ratings. Collapsing the two rating categories was justified by the high correlation between these two variables observed in the present sample [*r*(18) = 0.90, *p* < 0.001 for both ratings and RTs] and across samples (Dolcos et al., 2012).

### fMRI Data Acquisition, Preprocessing, and Analyses

Functional MRI data were recorded using a 3T Siemens Tim Trio scanner, and consisted of a series of T2^∗^-weighted images acquired axially, using an echo-planar sequence [repetition time (TR) = 2000 ms, echo time (TE) = 40 ms, field of view = 256 mm × 256 mm, number of slices = 28, voxel size = 4 mm × 4 mm × 4 mm, flip angle = 90°] (see also Supplementary Material S2). All preprocessing and statistical analyses of fMRI data were performed using SPM8 (Wellcome Department of Cognitive Neurology, London, United Kingdom). During preprocessing, fMRI data were first corrected for differences in acquisition time between slices for each image. Second, each functional image was spatially realigned to the first image of each run to correct for head movement. Third, these images were transformed into the standard anatomical space defined by the Montreal Neurological Institute (MNI) template implemented in SPM8. Finally, the normalized functional images were spatially smoothed using an 8 mm Gaussian kernel, full-width-at-half-maximum (FWHM), to increase the signal-to-noise ratio.

#### Analyses of Neural Response Linked to Observing Ingroup vs. Outgroup Host Behaviors and Handshake

At the first level, each participant’s preprocessed functional data were analyzed using an event-related design in the general linear model (GLM) framework. In keeping with the previous study using a similar paradigm (Dolcos et al., 2012), evoked hemodynamic responses to all events were modeled with a delta (stick) function corresponding to the onset of each event convolved with canonical hemodynamic response function. Our main GLM included regressors for different types of behavior (approach, avoidance, static) and handshake (handshake, no-handshake) as the events of interest, separately for ingroup and outgroup conditions. In addition, the onsets of rating screens as well as six motion parameters calculated during spatial realignment for each run were modeled as the events of no interest. These analyses generated contrast images identifying differential BOLD activation associated with observing each event of interest relative to baseline within each participant.

At the second level, the contrast images generated for each participant were entered into a series of ANOVAs using the flexible factorial design implemented in SPM8, in which a subject factor was included. To investigate the differences in BOLD response associated with the observation of (1) different non-verbal behaviors (approach, avoidance, static) as well as (2) handshake displayed by ingroup and outgroup hosts, a 3 (Behavior) × 2 (Host Race) and 2 (Handshake) × 2 (Host Race) ANOVA were conducted, respectively. Each of the ANOVA models produced three *F*-contrast maps identifying the regions showing the (1) main effect of behavior (or handshake), (2) main effect of host race, and (3) interaction effect between behavior (or handshake) and host race. To further characterize the significant main effects and interactions identified based on these *F*-contrast maps, a series of *post hoc t*-tests were performed. Specifically, whole-brain *t*-contrast maps were inclusively masked with the corresponding *F*-contrast maps to identify the directionality of activation within those clusters showing significant ANOVA main effects. Moreover, for those clusters showing significant interactions between behavior (handshake) and host race, mean parameter estimates (i.e., beta values) were extracted and plotted to clarify which condition(s) were driving the interaction.

#### Analyses of Functional Connectivity Linked to Observing Ingroup vs. Outgroup Host Behaviors

To further investigate modulation of functional connectivity involving brain regions identified by the above analyses of activation as showing a significant Behavior × Host Race interaction (see section “Results”), functional connectivity analyses were performed using the beta-series correlation method (Rissman et al., 2004; Gottlich et al., 2015). The seeds for connectivity analyses were defined as the peak voxels showing the strongest interaction effect in each functional cluster: ACC (Talairach coordinates: *x* = 0, *y* = 20, *z* = 21), mPFC (*x* = 4, *y* = 51, *z* = 20), and right superior frontal cortex (SFC) (*x* = 20, *y* = 3, *z* = 55).

At the first level, a GLM was created in which BOLD response time-locked to the onset of the host’s behavior was modeled using a canonical hemodynamic response function individually by a separate covariate, producing different parameter estimates for each trial and for each participant. The onsets of handshake and the rating period in each trial, and the six motion parameters for each run, were also included in this GLM. Next, seed-based correlations were calculated voxel-wise for each participant and for each of the six conditions of interest resulting from a Behavior × Host Race interaction. This procedure yielded an individual correlation map between each seed region and all other voxels in the brain separately for each condition of interest, which was normalized using Fisher’s *z* transformation. At the second level, these individual correlation maps were entered into a series of ANOVAs to identify voxels that showed changes in functional connectivity (based on trial-by-trial variability in parameter estimates) with each of the seed regions as a function of observing different types of social encounters with ingroup vs. outgroup hosts.

Correction for multiple comparisons was conducted using the updated version (August 2016) of the 3dFWHMx and 3dClustSim programs from the AFNI software package (Cox, 1996). In the present study, activity was investigated within a mask of *a priori* regions of interest (ROIs), similar to a procedure employed by previous studies of group membership (Telzer et al., 2015). Specifically, our ROI mask consisted of brain regions that have been previously implicated in the processing of group membership in various task contexts (Kubota et al., 2012; Molenberghs, 2013; Shkurko, 2013), along with those more generally involved in action observation and social cognition relevant for the present task (Dolcos et al., 2012). These regions included (bilaterally) the medial and lateral frontal cortex, cingulate cortex, amygdala, fusiform gyrus, insula, inferior parietal lobule, and pSTS with the surrounding lateral parietal/temporal/occipital regions (e.g., TPJ, extrastriate cortex). These ROIs were created based on the structures from the Automated Anatomical Labeling Atlas (Tzourio-Mazoyer et al., 2002) available as part of the WFU PickAtlas toolbox in SPM, with the exception of the lateral parietal/temporal/occipital regions, covering the pSTS and surrounding areas. Because these latter regions could not be precisely defined based on the anatomical boundaries, we used a functional mask from a previous study using a similar paradigm (Dolcos et al., 2012). Specifically, this functional mask identified bilateral posterior lateral temporal areas extending into the TPJ and extrastriate cortex showing greater activity to the observation of dynamic social interactions relative to static social scenes (at a height threshold of *p* < 0.05 corrected for false discovery rate). The resulting mask included 7,210 voxels. For a full description of the ROI mask, see Supplementary Material S1.

Results of 3dClustSim with 10,000 independent iterations indicated a voxel-wise height threshold of *p* < 0.005 and a minimum cluster extent threshold of 15 contiguous voxels (960 mm^3^) corresponding to *p* < 0.05, family-wise error corrected. This threshold is overall similar to the ones recently employed in studies of group membership and race (e.g., Cassidy and Krendl, 2016; Cassidy et al., 2016; Krendl and Kensinger, 2016).

#### Analyses of Brain-Behavior Interaction Linked to Observing/Evaluating Ingroup vs. Outgroup Social Encounters

Finally, to identify brain regions whose BOLD activation was related to individual variation in behavioral ratings and/or RTs, brain-behavior covariations were investigated by calculating across-participant covariations between the fMRI signals (i.e., parameter estimates) associated with observing ingroup/outgroup social encounters and the ratings/RTs for the relevant conditions. These analyses were restricted to the regions independently identified from the ANOVAs as showing sensitivity to different types of social encounters with ingroup and outgroup hosts. For each region, mean parameter estimates were extracted for each condition of interest and were submitted to bivariate correlation analyses to examine the relations between brain activity and behavioral measures in different conditions. Because significant differences in behavioral responses were identified only with respect to RTs for the interest ratings (see section “Behavioral Results”), analyses of brain-behavior relations also focused on the interest ratings as well as their RTs.

## Results

### Behavioral Results

#### Positive Impact of Approach Behaviors and Handshakes on Ratings of Social Encounters

**FIGURE 2:**
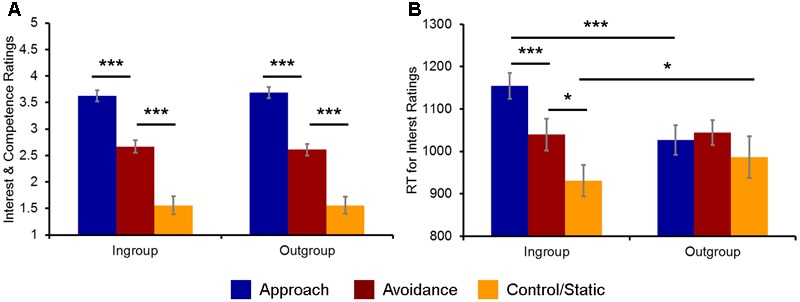
Behavioral indices of the evaluation of ingroup and outgroup social encounters. **(A)** Participants’ ratings of competence and interest for approach, avoidance, and static non-verbal behaviors were overall similar across ingroup and outgroup social encounters. **(B)** Participants’ RTs for evaluating their interest in doing business were significantly faster/slower for ingroup static/approach behaviors, respectively, compared to the corresponding outgroup conditions. ^∗^*p* < 0.05, ^∗∗∗^*p* < 0.001.

As expected, overall competence and interest ratings were highest for social encounters involving approach (*M* = 3.65, *SD* = 0.46), followed by avoidance (*M* = 2.63, *SD* = 0.52) and then by static (*M* = 1.56, *SD* = 0.75) displays of non-verbal behaviors, as confirmed by a 3 (Behavior) × 2 (Host Race) ANOVA yielding a significant main effect of Behavior: *F*(2,38) = 80.09, *p* < 0.001, $\eta_{p}^{2}$ = 0.81 (**Figure 2A**). *Post hoc t*-tests confirmed that the ratings for approach, avoidance, and static non-verbal behaviors were on average significantly different from each other [approach vs. avoidance: *t*(19) = 6.88, *p* < 0.001; approach vs. static: *t*(19) = 11.16, *p* < 0.001; avoidance vs. static: *t*(19) = 6.79, *p* < 0.001]. Neither the main effect of Host Race nor the interaction between Behavior × Host Race was significant [Host Race: *F*(1,38) = 0.04, *p* = 0.84, $\eta_{p}^{2}$ = 0.002; Behavior × Host Race: *F*(2,38) = 2.39, *p* = 0.11, $\eta_{p}^{2}$ = 0.11]. Moreover, the ratings for social encounters with a handshake (*M* = 3.44, *SD* = 0.45) were overall higher than those without it (*M* = 2.86, *SD* = 0.40), as revealed by a 2 (Handshake) × 2 (Host Race) ANOVA yielding a significant main effect of Handshake: *F*(1,19) = 31.89, *p* < 0.001, $\eta_{p}^{2}$ = 0.63. Again, neither the main effect of Host Race nor the interaction between Handshake × Host Race was significant [Host Race: *F*(1,19) = 0.01, *p* = 0.94, $\eta_{p}^{2}$ = 0.001; Behavior × Host Race: *F*(1,19) = 0.37, *p* = 0.55, $\eta_{p}^{2}$ = 0.02].

#### Modulation of RTs by Evaluating Different Social Encounters with Ingroup Hosts

Turning to the analyses of RTs, a 3 (Behavior) × 2 (Host Race) ANOVA first yielded a significant main effect of Behavior: *F*(2,38) = 7.65, *p* = 0.002, $\eta_{p}^{2}$ = 0.29. *Post hoc t*-tests revealed that RTs for evaluating social encounters involving approach (*M* = 1024.57, *SD* = 131.63) and avoidance behaviors (*M* = 1064.02, *SD* = 124.14) were on average significantly slower than those for evaluating static behaviors (*M* = 935.60, *SD* = 193.79) [approach vs. static: *t*(19) = 2.57, *p* = 0.02; avoidance vs. static: *t*(19) = 3.10, *p* = 0.006]. RTs for approach and avoidance behaviors were not significantly different from each other: *t*(19) = 1.45, *p* = 0.16. A main effect of Host Race was not significant: *F*(1,38) = 1.89, *p* = 0.19, $\eta_{p}^{2}$ = 0.09. However, an interaction between Behavior × Host Race was significant: *F*(2,38) = 4.73, *p* = 0.015, $\eta_{p}^{2}$ = 0.20.

Follow-up analyses revealed that the observed ANOVA interaction was driven by RTs for the interest ratings [*F*(2,38) = 18.94, *p* < 0.001, $\eta_{p}^{2}$ = 0.50], but not for the competence ratings [*F*(2,38) = 1.25, *p* = 0.30]. *Post hoc t*-tests confirmed that RTs for the interest ratings for approach (*M* = 1153.35, *SD* = 134.12), avoidance (*M* = 1039.42, *SD* = 169.08), and static (*M* = 927.25, *SD* = 163.33) behaviors by ingroup hosts were significantly different from each other [approach vs. avoidance: *t*(19) = 4.12, *p* < 0.001; approach vs. static: *t*(19) = 7.04, *p* < 0.001; avoidance vs. static: *t*(19) = 2.65, *p* = 0.016], whereas there were no significant differences in RTs between approach (*M* = 1026.86, *SD* = 154.32), avoidance (*M* = 1044.31, *SD* = 131.60), and static (*M* = 983.56, *SD* = 215.25) behaviors by outgroup hosts [approach vs. avoidance: *t*(19) = 0.69, *p* = 0.50; approach vs. static: *t*(19) = 0.96, *p* = 0.34; avoidance vs. static: *t*(19) = 1.41, *p* = 0.18] (**Figure 2B**).

Furthermore, direct comparisons of the ingroup vs. outgroup conditions for each type of behavior showed that RTs for the interest ratings for ingroup approach behaviors were significantly slower than those for outgroup approach behaviors [*t*(19) = 7.76, *p* < 0.001], whereas RTs for ingroup static behaviors was significantly faster than those for outgroup static behaviors [*t*(19) = 2.15, *p* = 0.04]. There were no significant differences in RTs between ingroup and outgroup avoidance behaviors [*t*(19) = 0.21, *p* = 0.84]. Finally, a 2 (Handshake) × 2 (Host Race) ANOVA did not yield a significant interaction between Handshake × Host Race: *F*(1,19) = 0.01, *p* = 0.57, $\eta_{p}^{2}$ = 0.001. Taken together, these behavioral findings partially confirm our first hypothesis and show that participants show bias toward their racial ingroup members in evaluating their behaviors in the context of business interactions, as reflected in faster/slower RTs for ingroup static/approach behaviors, respectively, compared to the corresponding outgroup conditions.

### fMRI Results

#### Observing Ingroup and Outgroup Dynamic Social Interactions and Handshakes Engages the Neural Networks Involved in Action Observation and Social Cognition

**FIGURE 3:**
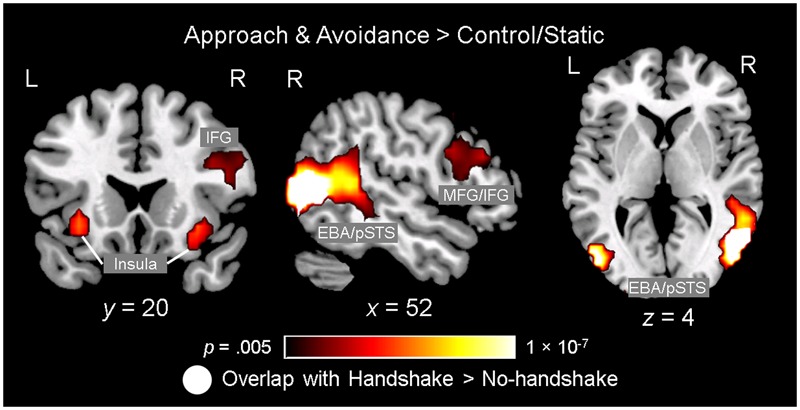
Observing ingroup and outgroup dynamic social interactions and handshakes engages the neural networks involved in action observation and social cognition. Observing approach and avoidance compared to static behaviors was associated with increased activity in a network of brain regions previously implicated in action observation and social cognition, including the lateral prefrontal cortex (PFC) regions [e.g., middle/inferior frontal gyri (MFG/IFG)], insula, left inferior parietal lobule (not shown), and bilateral temporo-occipital regions covering broader areas including the posterior superior temporal sulcus (pSTS) and extrastriate body area (EBA). Interestingly, an independent ANOVA examining the effect of observing handshakes also identified the right pSTS/EBA area showing significant spatial overlap with this right temporo-occipital area. Activation maps identifying the regions showing the effect of observing approach/avoidance behaviors relative to control (gradient), and the right pSTS/EBA area also showing the effect of observing handshake (white), are overlaid on top of a high-resolution anatomical template image in the MNI space.

Observing dynamic social interactions with ingroup and outgroup hosts, relative to static social scenes, engaged a network of brain regions implicated in action observation and social cognition. First, a 3 (Behavior) × 2 (Host Race) ANOVA identified a set of regions showing a main effect of Behavior. *Post hoc* analyses confirmed that the regions showing increased activity for observing dynamic social interactions than for static social scenes (i.e., approach and avoidance > control/static) consisted of the pSTS (bilaterally, with a rightward asymmetry, extending into the surrounding regions such as the superior/middle temporal gyrus and middle occipital gyrus/EBA), lateral PFC (bilaterally, covering both middle and inferior frontal gyri), left inferior parietal lobule, and posterior cingulate gyrus (see **Figure 3** and **Table 1**, and also Supplementary Material S3). For the contrast approach > avoidance, no region was identified as showing increased activity for observing approach than avoidance behaviors at the corrected threshold. For the contrast avoidance > approach, a cluster in the right fusiform gyrus extending into the adjacent extrastriate cortex as well as the left fusiform gyrus showed higher activity for observing avoidance than approach behaviors (see **Table 1**). Further exploratory examination of the contrast outside the *a priori* ROI mask revealed that the activation in the right fusiform area is actually part of a larger cluster extending into the cuneus (*x* = 16, *y* = -93, *z* = 8; BA 17), the peak of which was located close to the one observed in the initial Dolcos et al. (2012) study for the corresponding contrast (*x* = 8, *y* = -77, *z* = 11; BA 17).

**Table 1:** Brain regions showing the main effects of Behavior and Handshake in observing ingroup and outgroup social encounters.

Brain region	Side	BA	Talairach peak coordinates			*t*	Voxels	Volume (mm^3^)
			*x*	*y*	*z*			
**(A) Dynamic (approach and avoidance) > control/static**					
Lateral frontal cortex					
Inferior frontal gyrus	L	47	-28	23	-8	5.88	21	1344
Insula	L	13	-32	19	-1	5.70		
Middle frontal gyrus	R	6	36	3	55	5.86	184	11776
Middle frontal gyrus	R	46	51	28	21	4.49		
Inferior frontal gyrus	R	44	40	9	25	5.15		
Inferior frontal gyrus	R	47	32	23	-5	8.11	24	1536
Parietal cortex					
Superior parietal lobule	L	7	-32	-44	61	3.01	15	960
Inferior parietal lobule	L	40	-36	-40	46	3.39		
Cingulate gyrus	R	23	0	-14	30	6.07	17	1088
Lateral temporal/occipital cortex					
Superior temporal gyrus	L	22	-40	-54	14	4.98	150	9600
Middle temporal gyrus	L	39	-55	-66	11	8.70		
Middle occipital gyrus	L	19	-48	-77	8	10.10		
Middle occipital gyrus	L	18	-28	-97	1	5.08		
Middle temporal gyrus	R	39	51	-66	11	12.11	377	24128
Middle temporal gyrus	R	21	59	-46	10	8.68		
Inferior parietal lobule	R	40	63	-38	24	6.14		
**(B) Control/static > dynamic (approach and avoidance)**					
Frontal cortex					
Middle frontal gyrus	L	8	-24	29	39	6.00	76	4864
Occipital cortex								
Lingual gyrus	L	18	-24	-78	-6	7.79	40	2560
**(C) Approach > avoidance**					
No suprathreshold voxels were identified.					
**(D) Avoidance > approach**					
Occipital cortex					
Fusiform gyrus	L	37	-28	-47	-8	4.69	19	1216
Fusiform gyrus	L	19	-32	-66	-7	3.06		
Parahippocampal gyrus	R	19	28	-51	-4	8.95	17	1088
Fusiform gyrus	R	37	32	-43	-5	7.83		
Lingual gyrus	R	18	24	-74	-3	5.92	15	960
**(E) Handshake > no-handshake**					
Lateral temporal/occipital cortex					
Middle temporal gyrus	R	37	55	-62	7	5.43	96	6144
Middle occipital gyrus	R	19	51	-70	-3	3.66		
**(F) No-handshake > handshake**					
No suprathreshold voxels were identified.					

*The table identifies a network of brain regions showing main effects of behavior and handshake, as revealed by a 3 (Behavior) × 2 (Host Race) ANOVA and a 2 (Handshake) × 2 (Host Race) ANOVA, respectively. These *F*-contrast maps were used to inclusively mask the corresponding *T*-contrast maps to further identify the directionality of BOLD activation. All clusters reported in this table meet the significance threshold determined based on a Monte Carlo simulation, corrected for multiple comparisons at *p* < 0.05 (see section “Materials and Methods”). BA, Brodmann’s area; L, left; R, right.*

Second, a 2 (Handshake) × 2 (Host Race) ANOVA identified regions showing a main effect of Handshake. *Post hoc* analyses confirmed that the right pSTS/EBA region showed increased activity for observing handshake than the absence of it. Interestingly, this right pSTS/EBA cluster overlapped substantially with that identified in the separate ANOVA as showing increased activity for observing dynamic social interactions than static social scenes (**Figure 3**). No region was identified as showing a significant main effect of race. Taken together, these findings confirm our second hypothesis and show that observing dynamic social interactions and handshakes engages broader networks of regions previously implicated in action observation and social cognition, and replicate previous findings using a similar experimental design (Dolcos et al., 2012).

#### Greater mPFC Sensitivity to Observing Ingroup Social Encounters Linked to Positive Evaluations of Ingroup Approach Behaviors

**FIGURE 4:**
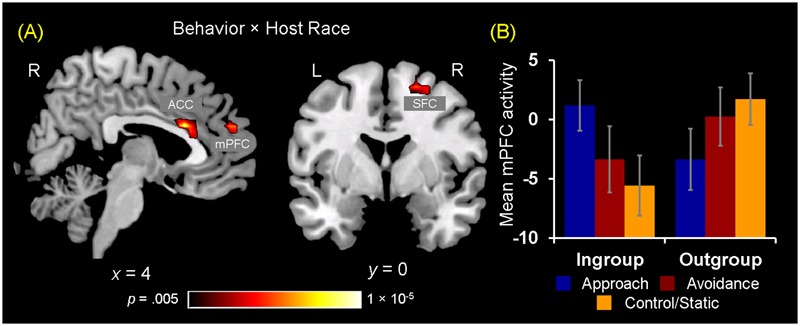
Brain regions showing differential activations to observing ingroup vs. outgroup social encounters. **(A)** A significant 3 (Behavior) × 2 (Host Race) ANOVA interaction effect was identified in a set of regions, including the medial prefrontal cortex (mPFC), anterior cingulate cortex (ACC), and right superior frontal cortex (SFC). **(B)** Mean parameter estimates extracted from the mPFC cluster showing a significant Behaivor × Host Race interaction revealed that the effect was primarily driven by significantly decreased activity for observing ingroup static behaviors compared to ingroup approach and outgroup static behaviors. These opposing patterns of activation linked to observing ingroup vs. outgroup social encounters were similarly observed for the ACC and SFC clusters (see Supplementary Figure S4B). Error bars indicate the standard error of the mean for each condition.

The two ANOVA models discussed above also identified a set of regions showing differential activation to observing ingroup vs. outgroup social encounters, as revealed by significant interaction effects between Behavior/Handshake × Host Race. First, a significant Behavior × Host Race interaction was observed in the right medial PFC (BA 9), ACC (BA 24/32), and right SFC (BA 6) (**Table 2** and **Figure 4A**, and also Supplementary Material S4). *Post hoc* analyses of mean parameter estimates extracted from each significant cluster for each condition of interest revealed that the observed interaction effect was driven by the opposing patterns of activation in these regions for the ingroup vs. outgroup conditions. Regarding the mPFC cluster, the pattern of its activation for the ingroup conditions mirrored the behavioral results discussed above, such that the mPFC activation was greatest for observing approach behaviors and was significantly reduced for observing static behaviors. The opposite pattern of activation was identified for observing outgroup behaviors (**Figure 4B**; see also Supplementary Material S4 for the patterns of activation in the ACC and SFC clusters). No region was identified as showing a significant main effect of race. Finally, an ANOVA with a covariate modeling RT for each condition also yielded similar results within the *a priori* ROI mask, and thus the observed results are not likely to reflect motor-related responses.

**Table 2:** Brain regions showing significant interaction effects of behavior/handshake and host race linked to observation of social encounters with ingroup and outgroup hosts.

Brain region	Side	BA	Talairach peak coordinates			*F*	Voxels	Volume (mm^3^)
			*x*	*y*	*z*			
**(A) Behavior × Host Race interaction**					
Frontal cortex					
Superior frontal gyrus	R	6	20	3	55	7.31	31	1984
Superior frontal gyrus	R	8	24	22	50	9.04		
Medial frontal gyrus	R	9	4	51	20	9.30	22	1408
Cingulate gyrus	L/R	24	0	20	21	12.98	50	3200
Cingulate gyrus	L	32	-12	21	28	12.23		
**(B) Handshake × Host Race interaction**					
Lateral temporal/occipital cortex					
Superior/middle temporal gyrus	R	39	40	-57	25	17.40	18	1152

*The table identifies brain regions showing the significant interaction effect of behavior and host race as revealed by a 3 (Behavior) × 2 (Host Race) ANOVA and, that of handshake and host race as revealed by a 2 (Handshake) × 2 (Host Race) ANOVA. All clusters reported in this table meet the significance threshold determined by a Monte Carlo simulation, corrected for multiple comparisons at *p* < 0.05 (see section “Materials and Methods”). BA, Brodmann’s area; L, left; R, right.*

**FIGURE 5:**
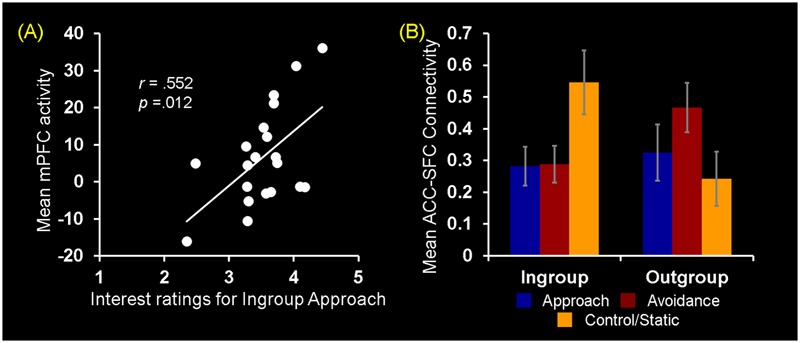
Neural ingroup bias as reflected in brain-behavior covariation and functional connectivity. **(A)** Brain-behavior covariation analyses identified a significant positive association between differential mPFC activity for ingroup approach vs. static behaviors (*y*-axis) and the interest ratings for ingroup approach behaviors (*x*-axis). A similar but slighltly weaker relationship was also identified by using the average of interest and competence ratings [*r*(18) = 0.496, *p* = 0.026]. **(B)** Analyses of whole-brain functional connectivity using as a seed the peak voxel (*x* = 0, *y* = 20, *z* = 21) of the ACC cluster shown in **Figure 4A** identified the right SFC region showing a significant Behavior × Host Race ANOVA interaction. *Post hoc* analyses revealed that the ACC-SFC connectivity significantly increased for observing ingroup static compared to ingroup approach [*t*(19) = 2.65, *p* = 0.02] and avoidance [*t*(19) = 2.84, *p* = 0.01] behaviors. In contrast, there was no difference in connectivity between the two regions for observing outgroup social encounters. Values on the *y*-axis represent the magnitude of correlation (*Z*-values) between activity in the ACC seed region and mean activity within the right SFC cluster calculated on the basis of trial-by-trial variability for each participant. Error bars indicate the standard error of the mean for each condition.

Turning to the results of brain-behavior covariation analyses, a significant relation was identified between increased mPFC activity for observing ingroup approach relative to static behaviors and the interest ratings. Specifically, those participants who showed increased activity in the mPFC for ingroup approach than static behaviors also rated their interest in doing business with ingroup members displaying approach behaviors more positively [*r*(18) = 0.552, *p* = 0.012] (**Figure 5A**). No significant covariation was identified between mPFC activity and RTs for the interest ratings. Additionally, no significant covariation was identified with activity in the ACC and right SFC clusters. Overall, these findings partially confirm our third hypothesis regarding the dissociable neural correlates of observing ingroup vs. outgroup social encounters.

#### Greater ACC-SFC Connectivity Linked to Processing of Static Non-verbal Display by Ingroup Hosts

Following identification of the mPFC, ACC, and SFC regions showing differential activations to ingroup vs. outgroup social encounters, analyses of functional connectivity were performed as 3 (Behavior) × 2 (Host Race) ANOVAs using as seeds peak activity of these regions independently identified from the activation analyses. First, a main effect of Behavior was identified using the mPFC and ACC seeds. Regarding the mPFC, *post hoc* analyses revealed that this region showed increased connectivity with the left pSTS/EBA and SFC for observing dynamic social interactions than static social scenes. Regarding the ACC, this region showed increased connectivity with bilateral pSTS areas and the right middle/inferior frontal gyrus for observing avoidance behaviors than approach and static behaviors (see Supplementary Material S5). No region was identified as showing a significant main effect of race. Second, regarding the ACC seed, a significant interaction between Behavior × Host Race was identified in the right SFC (*x* = 24, *y* = -1, *z* = 52). Interestingly, this right SFC cluster partially overlapped with the right SFC independently identified as showing a significant Behavior × Host Race interaction from the activation analyses (*x* = 20, *y* = 3, *z* = 55). *Post hoc* analyses showed that the ACC-SFC connectivity was significantly greater for observing ingroup static than ingroup approach and avoidance behaviors, whereas no difference was identified in the ACC-SFC connectivity within the outgroup conditions (**Figure 5B**). Taken together, these findings partially confirm our fourth hypothesis and show that neural ingroup bias linked to observing social encounters also manifest as reduced (and coordinated) activation in brain regions that may be involved in conscious regulatory processes.

#### Greater pSTS Sensitivity to Observing Handshakes with Ingroup Members

**FIGURE 6:**
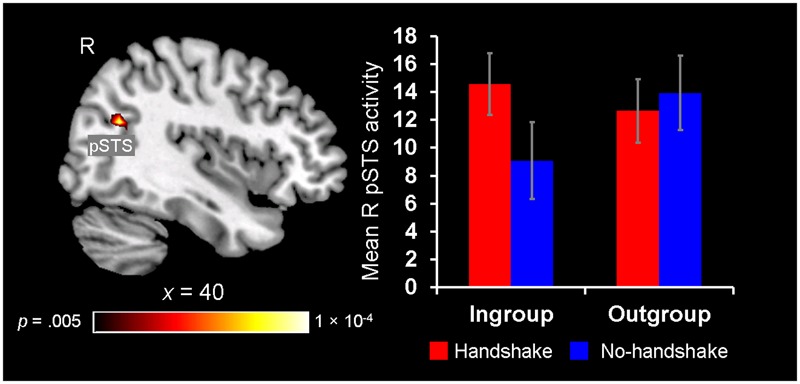
Greater pSTS sensitivity to observing handshakes with ingroup members. A significant 2 (Handshake) × 2 (Host Race) ANOVA interaction was identified in a set of regions, including the right posterior superior temporal sulcus (pSTS) **(left)**. *Post hoc* analyses of mean parameter estimates extracted from this right pSTS cluster revealed that this region showed greater activation for observing handshakes with ingroup hosts [*t*(19) = 3.00, *p* = 0.008], whereas its activity did not differentiate between the handshake and no-handshake conditions with outgroup hosts [*t*(19) = 1.20, *p* = 0.25] **(right)**. Error bars indicate the standard error of the mean for each condition.

Finally, neural ingroup bias was also identified in the context of observing handshakes, as revealed by the right pSTS showing a significant Handshake × Host Race interaction. *Post hoc* analyses revealed that the right pSTS showed increased activity to the presence of handshakes with ingroup members, whereas its response did not differentiate between the outgroup handshake vs. no-handshake conditions (**Figure 6**). These findings confirm and lend further support to our third hypothesis by showing that activity in the pSTS is sensitive to dynamic non-verbal behaviors displayed by ingroup members during social interaction, particularly when the behavior is expected as a common greeting behavior in one’s own culture (i.e., handshake).

## Discussion

Substantial changes in the racial/ethnic composition of the United States population emphasize the need to better understand the mechanisms involved in social interactions with racially ingroup and outgroup members. Extending the previous evidence in the literature, the present study identified findings that shed light on the neural correlates of racial ingroup bias linked to the observation of whole-body non-verbal behaviors in a defined social context. These findings will be discussed in turn below.

### Positive Impact of Approach Behaviors and Handshakes on Ratings of Social Encounters

Decades of research on intergroup bias have demonstrated that preferential positivity toward one’s ingroup members forms the basis of intergroup cognition, and could manifest independently of outgroup hostility (Brewer, 1999; Dovidio and Gaertner, 2010). Available evidence suggests that the processing of ingroup members tends to occur more spontaneously and effortlessly than that of outgroup members (Bernstein et al., 2007; Adams et al., 2010). Such spontaneous categorization of ingroup vs. outgroup members may allow one to cultivate a sense of social belonging, which in turn promotes one’s chance of survival and leads to a positive self-concept (Tajfel and Turner, 1986; Brewer, 1999). Based on these notions, we expected that participants would show bias toward their racial ingroup members in evaluating their behaviors in the context of business interactions, as possibly reflected in the ratings and/or RTs.

Regarding the ratings of business competence and interest, the present study did not identify significant differences by racial group membership at a group level. One possibility is that explicit ratings of social encounters may not be influenced by whether or not such encounters involve racially ingroup or outgroup members. However, as discussed below, a significant relation was identified between the ratings of business interest with ingroup members and activity in a specific brain region previously implicated in neural ingroup bias (Molenberghs, 2013). This suggests that ingroup bias in explicit ratings of non-verbal social encounters may be more likely to manifest as a function of individual variation in neural responses possibly linked to the processing of ingroup members.

### Modulation of RT by Evaluating Different Social Encounters with Ingroup Hosts

Regarding RTs, the present study identified differences in RTs for evaluating social encounters with ingroup vs. outgroup members, which were driven by slower RTs for ingroup than outgroup approach behaviors, and faster RTs for ingroup than outgroup static behaviors. These findings are overall consistent with previous evidence for greater sensitivity to non-verbal cues displayed by ingroup members compared to those displayed by outgroup members (Elfenbein and Ambady, 2002; Adams et al., 2010), and this enhanced sensitivity might have resulted in greater differentiation in RTs between ingroup approach, avoidance, and static behaviors. More specifically, slower RTs for ingroup than outgroup approach behaviors may reflect greater interest in ingroup members conveying positive emotions. This is consistent with previous evidence showing that participants spent more time viewing pictures of racial ingroup than outgroup members displaying pleasant emotions, while they did not show such difference by group membership in viewing unpleasant pictures (Brown et al., 2006). In contrast, faster RTs for ingroup than outgroup static behaviors may reflect more spontaneous processing of ingroup members (Bernstein et al., 2007; Van Bavel et al., 2008; Senholzi and Ito, 2013).

### Observing Ingroup and Outgroup Dynamic Social Interactions and Handshakes Engages the Neural Networks Involved in Action Observation and Social Cognition

Replicating the previous findings (Dolcos et al., 2012), observing dynamic social interactions compared to static social scenes engaged a broad network of regions associated with action observation and social cognition, including the lateral temporo-occipital regions (e.g., pSTS/EBA), lateral PFC, inferior parietal lobule, and posterior cingulate gyrus. Moreover, an independent analysis examining differential activation linked to observing handshakes also showed that a subregion of the larger lateral temporo-occipital cluster showing increased activity for observing approach/avoidance behaviors also showed greater activity for observing handshakes. This region, broadly encompassing BAs 19 and 37, shows significant spatial overlap with regions previously identified as likely showing activations in studies of “*action observation*” (Yarkoni et al., 2011), and also in processing dynamic emotional bodily expressions (Peelen et al., 2007). Taken together, the present findings suggest that the right pSTS/EBA and its surrounding regions may be generally involved in processing of dynamic non-verbal bodily signals, including greeting behaviors preceding social interaction in a business context.

Regarding differential activations in observing approach and avoidance behaviors, increased activity for observing avoidance than approach behaviors was identified in some occipital regions (fusiform gyrus, extrastriate cortex, cuneus). This is consistent with the previous findings identifying similarly increased activity in the cuneus for avoidance than approach behaviors (Dolcos et al., 2012). However, unlike the Dolcos et al. (2012) study, the present study did not identify regions (e.g., pSTS) showing increased activity for observing approach than avoidance behaviors. This may be related to differences in the experimental paradigms between the two studies, including host characteristics (i.e., race, gender) as well as the number of trials, that might have affected the overall salience of non-verbal behaviors displayed by the hosts.

### Greater mPFC Sensitivity to Observing Ingroup Social Encounters Linked to Positive Evaluations of Ingroup Approach Behaviors

Extending prior evidence for the neural correlates of ingroup vs. outgroup processing, the present study identified an area in the mPFC showing opposing patterns of activation linked to observing social encounters with ingroup vs. outgroup members. Several studies have demonstrated that the mPFC shows increased activity when processing information about ingroup than outgroup members, thus linking this region to neural ingroup bias in various social contexts (Cunningham and Van Bavel, 2009; Freeman et al., 2010; Morrison et al., 2012; Molenberghs and Morrison, 2014). In the present study, the interaction concerning the mPFC was driven particularly by its decreased activity for observing ingroup static behavior, which was significantly increased for ingroup approach and outgroup static behaviors. One possible explanation for this finding is that mPFC activation reflects a degree of elaborate evaluative processes engaged while observing social encounters with ingroup and outgroup members.

Although the mPFC is one of the major nodes part of the default network that typically shows deactivation during cognitively effortful tasks, significant activation of this region has been consistently reported in relation to self-referential processing (Gusnard et al., 2001), inference of others’ mental states (i.e., mentalization; Amodio and Frith, 2006), and integration of social knowledge (Van Overwalle, 2009). As discussed above, given that processing of ingroup members may be relatively more spontaneous in general, it is possible that evaluations of ingroup static behaviors required least mentalization, and hence was associated with decreased activity in the mPFC. This is also consistent with our findings regarding increased functional connectivity between mPFC and pSTS/EBA for observing approach and avoidance than static behaviors at a general level, possibly suggesting reduced interaction between regions involved in action understanding and mentalization when observing static behaviors.

Ingroup approach behaviors, however, may be of particular significance and interest in the context of observing social encounters (Tajfel and Turner, 1986; Brewer, 1999). Processing of ingroup approach behaviors may therefore involve enhanced integration of self-knowledge with social judgments and goals, possibly resulting in more positive evaluations of approach behaviors. This interpretation is consistent with our behavioral results identifying slowest RTs for evaluating ingroup approach behaviors, and also with previous evidence identifying a similar anterior mPFC region showing greater activity for ingroup than outgroup members in the context of individuated (vs. superficial) judgments (Freeman et al., 2010). Additionally, observation of outgroup static behaviors may also require greater engagement of mentalization and evaluative processes, possibly due to unfamiliarity or uncertainty associated with encounters with outgroup members (Brewer, 1999). This may have resulted in increased mPFC activity along with slower RTs in evaluating outgroup compared to ingroup static behaviors.

Alternatively, it is also possible that these effects, especially linked to the observation of ingroup approach behaviors, may partially reflect the processing of more basic (non-social) emotional stimuli. For instance, previous studies have identified greater mPFC activity linked to the processing of appetitive/positive vs. aversive/negative stimuli (Wager et al., 2003; Lang and Bradley, 2010), consistent with the idea that this region is part of the mesocorticolimbic reward circuit in the brain (Tzschentke, 2000). Therefore, it is possible that greater mPFC activity associated with observing Caucasian approach behaviors is not unique to the processing of ingroup stimuli *per se*, but in part reflects responses linked to the processing of general positive stimuli in the current task context. This idea may also explain previous evidence identifying the so-called “outgroup favoritism,” in which individuals belonging to socially disadvantaged groups (e.g., African–American) showed implicit preference toward their outgroup members (e.g., Caucasian), by unconsciously endorsing social stereotypes about their ingroup (Dasgupta, 2004; Ashburn-Nardo and Johnson, 2008; see also Jost and Banaji, 1994). Given that the current sample only consisted of Caucasian individuals, future studies should examine racially diverse subject samples to clarify this aspect further.

Taken together, the present findings regarding the mPFC confirm the role of this region in preferentially processing information about ingroup members (or general positive stimuli in the present task context), and further suggest that its response may uniquely scale with the degree of elaborate evaluative processes engaged during observation of social encounters with ingroup members/Caucasian stimuli.

### Greater ACC-SFC Connectivity Linked to Processing of Static Non-verbal Display by Ingroup Hosts

In the context of processing ingroup vs. outgroup members, the involvement of the ACC and SFC (e.g., dlPFC) has been primarily linked to deliberate regulation of automatic biases associated with racial outgroups (Bartholow and Henry, 2010). Based on the previous evidence, one possible explanation for the present finding is that activity in the ACC and SFC reflects the degree of cognitive control and regulatory processes engaged during observation of social encounters. In this context, engagement of these regions may be assumed minimal while observing ingroup static behaviors, which may be processed most spontaneously among all the conditions. This interpretation is further supported by the results of our functional connectivity analyses, demonstrating greater functional connectivity between the ACC and SFC areas during observation of ingroup static compared to approach/avoidance behaviors. Interestingly, the ACC-SFC functional connectivity was not modulated by the type of social encounters with outgroup members. This suggests that the present findings regarding functional connectivity between the ACC and SFC can be better explained as a form of ingroup bias as opposed to outgroup bias.

Taken together, the present findings suggest that coordinated yet decreased activity in the ACC and SFC may reflect reduced monitoring and/or regulatory processes during observation of ingroup static behaviors. Such mechanisms may allow adaptive reallocation of resources for processing other stimuli that may require top-down control-related processes for further evaluations. This interpretation is consistent with the present findings identifying increased functional connectivity at a general level between the ACC and regions including bilateral pSTS for observing avoidance than approach and static behaviors, possibly suggesting greater engagement of action monitoring when observing and evaluating the hosts displaying avoidance behaviors.

### Greater pSTS Sensitivity to Observing Handshakes with Ingroup Members

Finally, the right pSTS region showing sensitivity to the presence of handshakes, specifically with ingroup members, is consistent with previous evidence linking similar brain regions to mentalization and reorientation of attention to salient stimuli (Decety and Lamm, 2007; Van Overwalle, 2009; Freeman et al., 2010), along with enhanced decoding of ingroup non-verbal behaviors (Adams et al., 2010). Therefore, one possibility is that increased activity in the right pSTS region linked to observing ingroup handshakes reflects greater mentalizing and attentional processing, possibly due to enhanced social and motivational significance associated with handshakes with racially ingroup members. Handshaking is a form of greeting behavior commonly practiced in Western cultures (Singh et al., 1998), which may influence impression formation and evaluative processes in the context of social encounters particularly among Caucasian individuals. Overall, our findings expand the current evidence regarding the involvement of the pSTS in social cognition, including its sensitivity to ingroup membership, and suggest that this region shows preferential activation for observing a culturally familiar greeting behavior with ingroup members.

Interestingly, overall the present study identified no significant main effect of race across analyses. This is consistent with evidence from a recent study examining the role of racial group membership in the perception and categorization of affective body postures (Watson and de Gelder, 2017). Specifically, the authors found that modulation of neural responses by race mostly emerged as an interaction of race and valence, such that outgroup effects were overall driven by positive emotions, whereas ingroup effects were driven more by negative emotions (Watson and de Gelder, 2017). These findings, together with the fact that no main effect of race was identified in the present study, point to the importance of considering the valence of non-verbal behaviors when examining the role of racial group membership in the context of social encounters.

### Limitations and Future Directions

The following limitations of the present study should be acknowledged. First, the present study did not explicitly focus on the impact of specific outgroup races (e.g., European–Americans vs. African–Americans), as commonly examined in previous studies of the neural correlates of race processing (Kubota et al., 2012). Because the present goal was to identify possible biases linked to general ingroup vs. outgroup differences, we employed stimuli that reflect the overall racial diversity of the local population. As a result, differences in the proportions of various racial groups made it difficult to obtain similar statistical power across different trials, based on host race. Nevertheless, the present study provides important initial evidence that will allow identification of potential biases in dynamic social interactions associated with specific races (Gutsell and Inzlicht, 2010), in future studies.

Second, similar to previous fMRI studies of social interaction (e.g., Schilbach et al., 2006; Pitskel et al., 2011; Zucker et al., 2011), the present study utilized the animated stimuli which enabled manipulations of whole-body non-verbal behaviors with a high degree of experimental control. However, it is possible that movies illustrating social encounters with real people may be associated with greater salience of group membership as well as greater social relevance for participants (de Borst and de Gelder, 2015). Therefore, future studies examining the neural correlates of ingroup bias in social evaluations may benefit from using such stimuli depicting social interactions between real humans (Huis in ’t Veld and de Gelder, 2015; Van den Stock et al., 2015b). Additionally, future studies may also benefit from the inclusion of dynamic “neutral” non-verbal behavior conditions (e.g., Kret et al., 2011) or an alternative static/control condition involving non-responsive characters rather than cardboard cutouts. The static social scene condition in the present study was justified by the fact that it simulates real-life contexts in which the human presence is replaced by similar cardboard images (e.g., of popular people or an organization’s employees), such as those posted in stores or banks. However, alternative neutral/control conditions as mentioned above may allow for identification of neural activity associated with observing approach and avoidance behaviors at a finer level, and of the role of racial group membership in these mechanisms.

Third, while the focus of the present study was on social encounters in a business context, the role of different types of social contexts remains unclear. Previous studies have shown that categorization of individuals from different racial groups can be affected by the social context in which their faces are processed (Freeman et al., 2013a,b). Moreover, there is also evidence showing that categorization of emotional bodily expression was facilitated when its valence was congruent with that of the background social scene (Kret and de Gelder, 2010). These findings suggest that the categorization and evaluation of ingroup vs. outgroup members may be differentially influenced by whole-body non-verbal behaviors depending on the specific context (e.g., formal/professional vs. informal/casual). The role of different social contexts should be explored further in future studies of non-verbal evaluations.

Fourth, although the interpretation of the observed effects regarding ACC-SFC connectivity as reflecting control-related processes is consistent with the role of these regions identified previously, the extent to which such processes were actually engaged during the present task remains unclear. Therefore, it would be important in future studies to clarify this by using experimental tasks that demand more clearly control-related processes in a defined social context. For instance, a task that involves both components of intra-/intergroup social evaluation and cognitive control (e.g., emotion regulation, working memory) may be informative in better understanding the role of ACC and SFC in the processing of group membership.

Finally, aside from these limitations related to the experimental design, it is also important to acknowledge the possible role of individual differences, particularly with respect to sex/gender and age. Notably, using an experimental paradigm similar to the one in the present study, we have recently shown that the effect of handshakes on the evaluation of social interactions is uniquely influenced by the sex/gender composition of interaction partners among Caucasian men (Katsumi et al., 2017). These findings, along with evidence pointing to gender differences in automatic ingroup bias (Rudman and Goodwin, 2004), warrant future investigations of sex/gender differences in the neural correlates of racial ingroup bias. In addition, available evidence suggests that advancing age is associated with increased difficulty in recognition of some non-verbal expressions (Ruffman et al., 2008). Interestingly, aging was also associated with greater implicit racial bias, linked to relative impairment in the regulation of automatic associations as a function of age (Gonsalkorale et al., 2009). These results render age differences particularly an interesting topic to examine in the context of the present task, given also that at least in some contexts aging appears to be associated with enhanced spontaneous regulation of emotional responses (Dolcos et al., 2014).

## Conclusion

Despite these limitations, the present investigation makes novel contributions to the literatures on the neural correlates of intergroup processes and non-verbal perception. By using an experimental paradigm involving observation of whole-body ingroup vs. outgroup social encounters in a defined context, the present study sheds light on how ingroup bias manifests both at the level of behavioral and neural responses. Evidence provided by behavioral assessments reveals ingroup bias as reflected in faster/slower RTs when evaluating ingroup static/approach behaviors, respectively. Mirroring these behavioral results, fMRI results identified ingroup biases in observing different types of social encounters, which were reflected in differential responses in the mPFC, ACC, SFC, and pSTS. Activity in the mPFC and pSTS possibly reflects greater mentalization associated with the processing of approach behaviors and handshakes, respectively, whereas greater functional connectivity between ACC and SFC suggests possibly more spontaneous processing of ingroup members’ static display of non-verbal behavior. These findings advance our understanding of the mechanisms underlying the processing of racial group membership in the context of non-verbal social encounters, and have implications for understanding factors that may lead to successful interactions with individuals from diverse racial groups.

## Ethics Statement

This study was carried out in accordance with the recommendations of the Institutional Review Board at the University of Illinois with written informed consent from all subjects. All subjects gave written informed consent in accordance with the Declaration of Helsinki. The protocol was approved by the Institutional Review Board at the University of Illinois.

## Author Contributions

SD designed the experiment and collected data. YK assisted with the stimulus creation, analyzed the data, and interpreted the results under SD’s supervision. YK and SD wrote the manuscript.

## Conflict of Interest Statement

The authors declare that the research was conducted in the absence of any commercial or financial relationships that could be construed as a potential conflict of interest.

## Footnotes

1 http://poser.smithmicro.com/poser.html
