# Supplementary material for: Neural Correlates of Racial Ingroup Bias in Observing Computer-Animated Social Encounters
Source: Front Hum Neurosci. 2018 Jan 4;11:632. doi: 10.3389/fnhum.2017.00632 (PMC5758503; doi:10.3389/fnhum.2017.00632)
Supplement: Supplementary file 1 [file Data_Sheet_1.DOCX]

Supplemental Material: Katsumi & Dolcos

Note: In all figures included in this documnt, the labels indicate MNI coordinates, and 3D functional/mask images are displayed on the ch2better.nii template available in MRIcron (<http://people.cas.sc.edu/rorden/mricron/index.html>).

**S1. Description of the a priori ROI mask**

The a priori ROI mask used in the present study consisted of brain regions that have been previously implicated in the processing of group membership in various task contexts ([Kubota, Banaji, & Phelps, 2012](#_ENREF_2); [Molenberghs, 2013](#_ENREF_3); [Shkurko, 2013](#_ENREF_5)), along with those more generally involved in action observation and social cognition relevant for the present task ([Dolcos, Sung, Argo, Flor-Henry, & Dolcos, 2012](#_ENREF_1)) (see Figure S1 below, and also Methods, pp. 8-9). All ROIs were created based on the structures from the Automated Anatomical Labeling Atlas ([Tzourio-Mazoyer et al., 2002](#_ENREF_6)) available in SPM, with the exception of the lateral parietal/temporal/occipital regions (covering the pSTS and surrounding areas), which were defined based on a functional mask from a previous study using a similar paradigm ([Dolcos et al., 2012](#_ENREF_1)). The resulting mask included 7,210 voxels (one voxel = 4^3^ mm^3^).


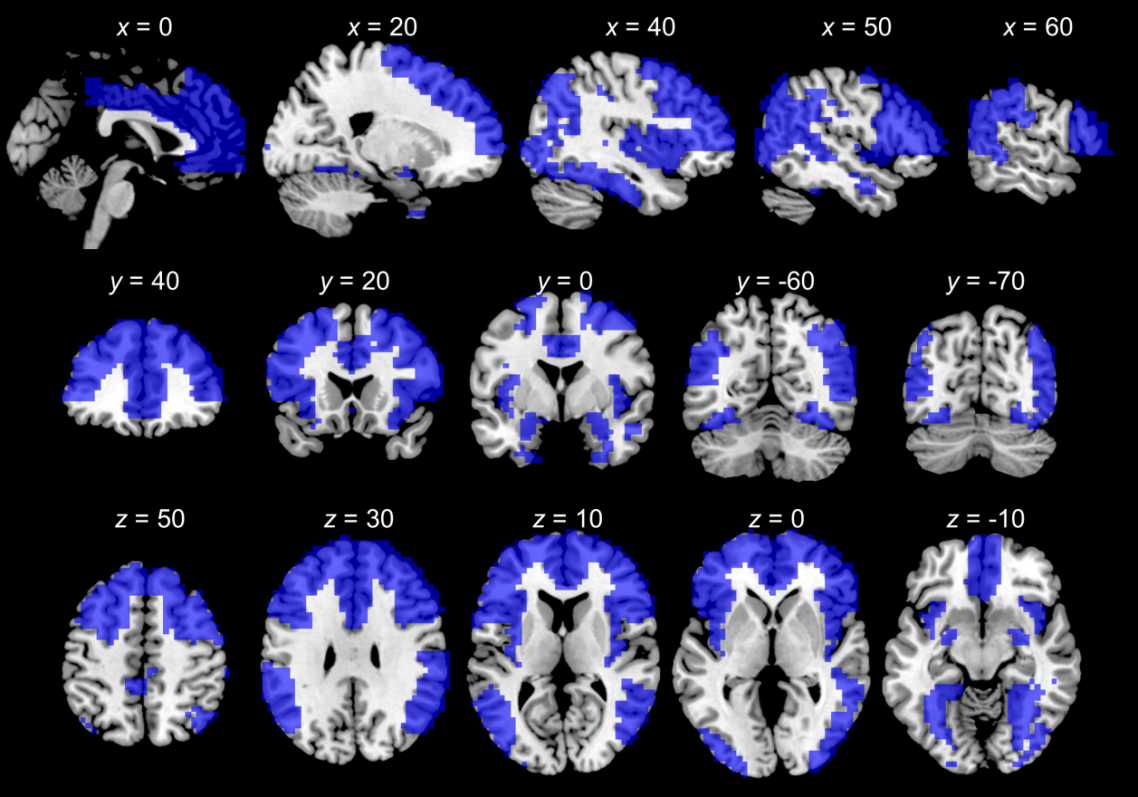


Figure S1. Coverage of the a priori ROI mask.

**S2. Coverage of EPI images**

The implicit mask image provided by SPM8 for the group-level random-effects analysis showing the coverage of the EPI images is shown below. The figure confirms that the whole brain was covered, with the exception of small parts of ventral frontal and middle temporal regions, which tend to be susceptible to artifacts due to the presence of air-pockets nearby. Notably, the coverage of the EPI images in the present study is very similar to that of a previous study examining the neural correlates of ingroup processing ([Morrison, Decety, & Molenberghs, 2012](#_ENREF_4)).


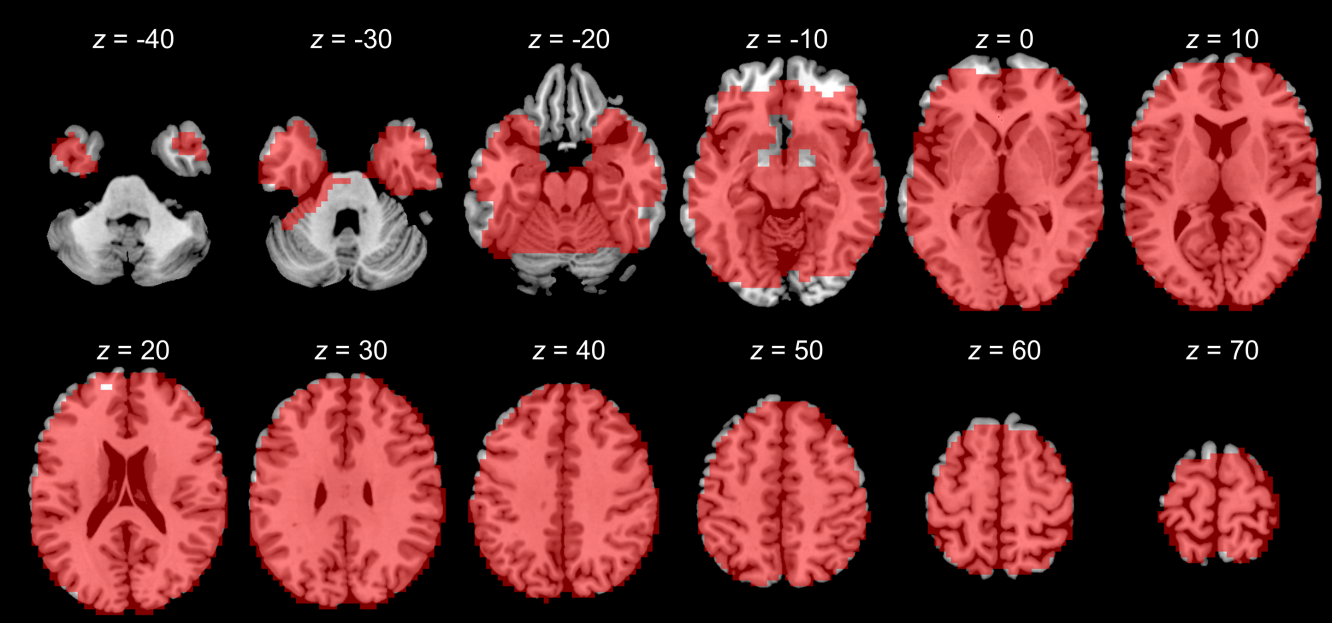


Figure S2. Coverage of EPI images.

**S3. Supplement to Figure 3**

**
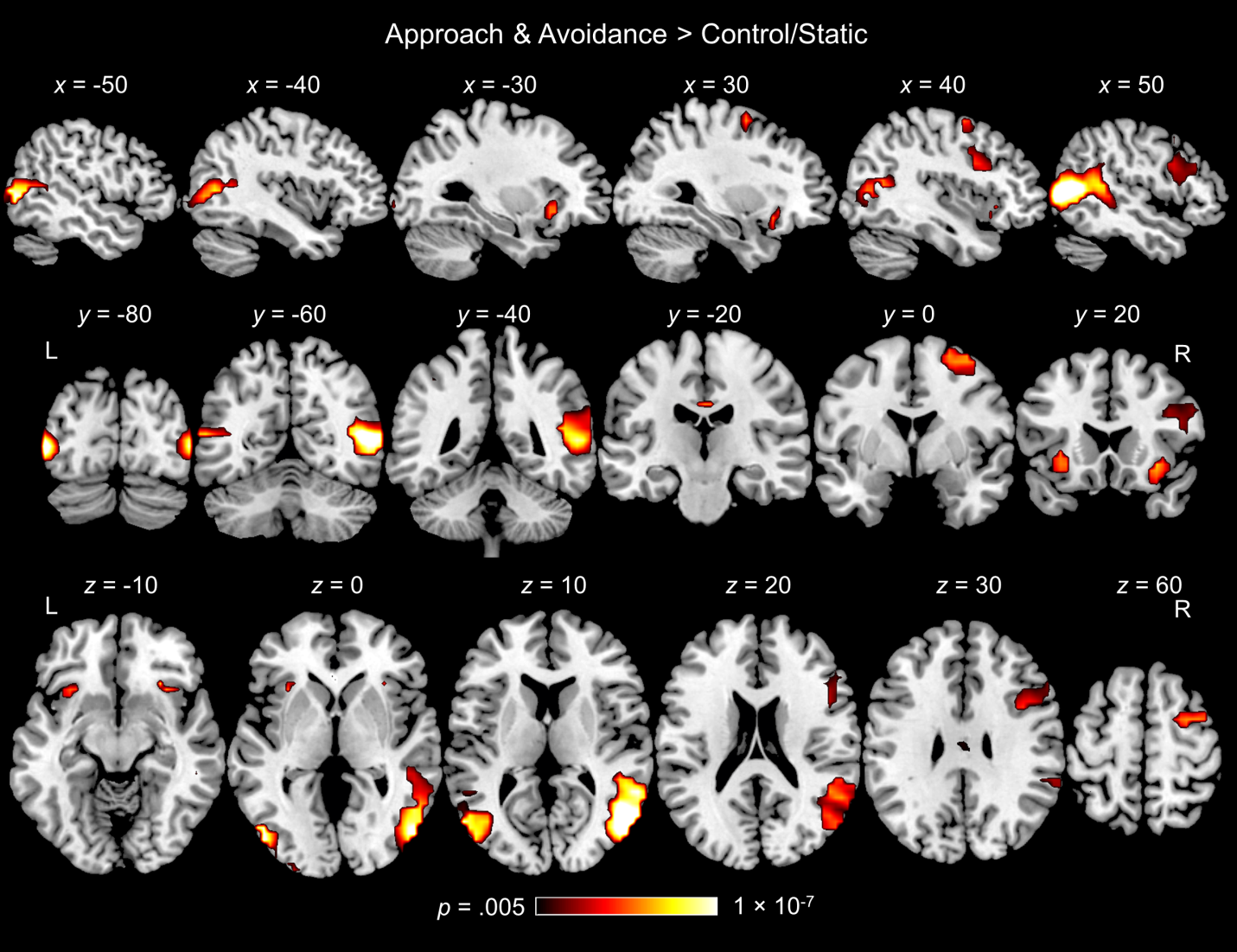
**

Figure S3(A). Brain regions showing greater activation for observing dynamic social interactions than static social scenes. This figure identifies brain regions showing greater activation for observing dynamic social interactions (approach and avoidance behaviors) than control/static social scenes, as revealed by a 3 (Behavior) × 2 (Host Race) ANOVA yielding a significant main effect of Behavior within the a priori ROI mask. The *F*-contrast map was used to inclusively mask the corresponding *T*-contrast map (i.e., Approach & Avoidance > Control/Static).


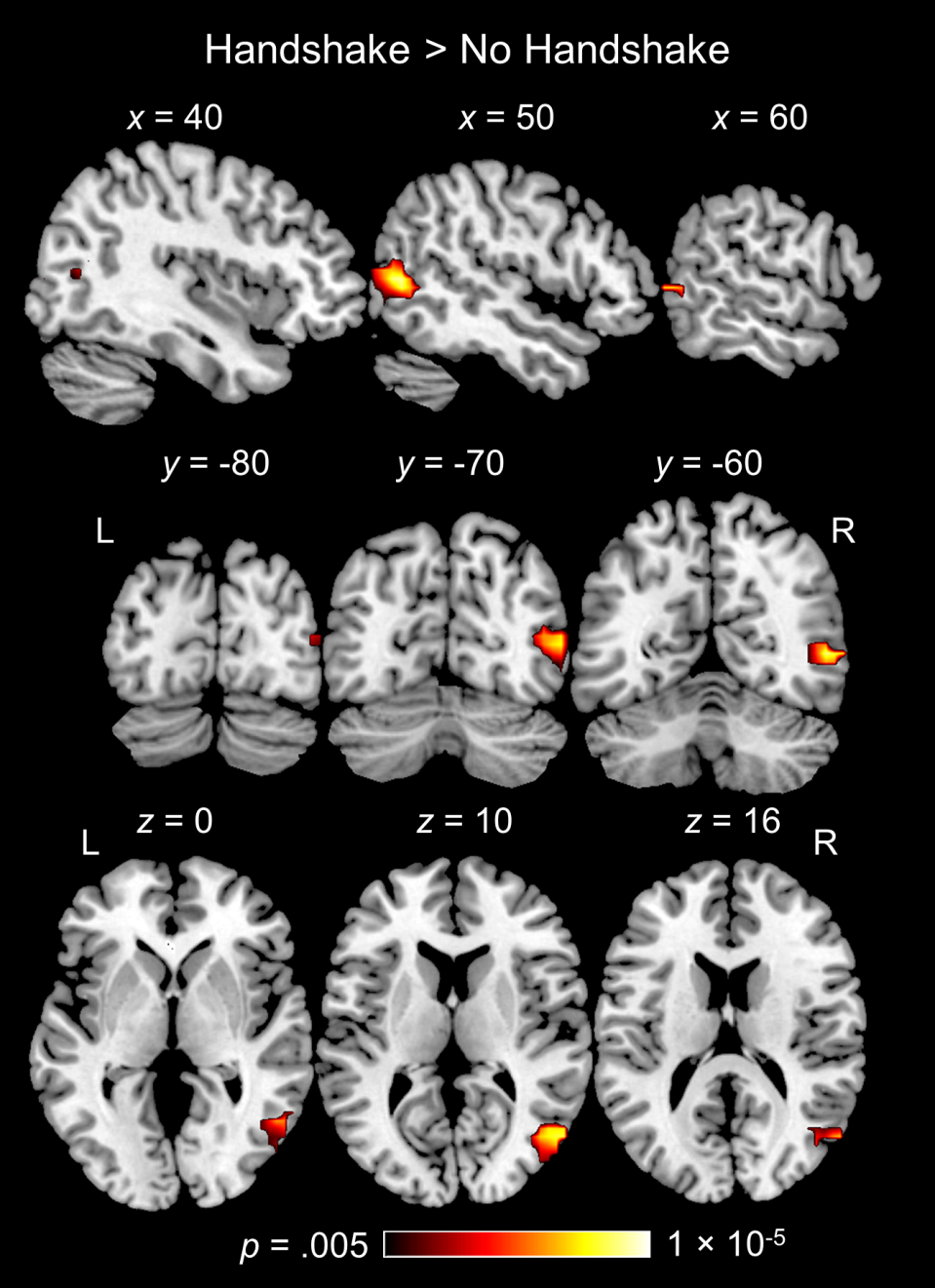


Figure S3(B). Brain regions showing greater activation for observing handshakes. This figure identifies brain regions showing greater activation for observing handshake than the absence of it during social interaction, as revealed by a 2 (Handshake) × 2 (Host Race) ANOVA yielding a significant main effect of Handshake within the a priori ROI mask. The *F*-contrast map was used to inclusively mask the corresponding *T*-contrast map (i.e., Handshake > No Handshake).

**S4. Supplement to Figure 4.**

**
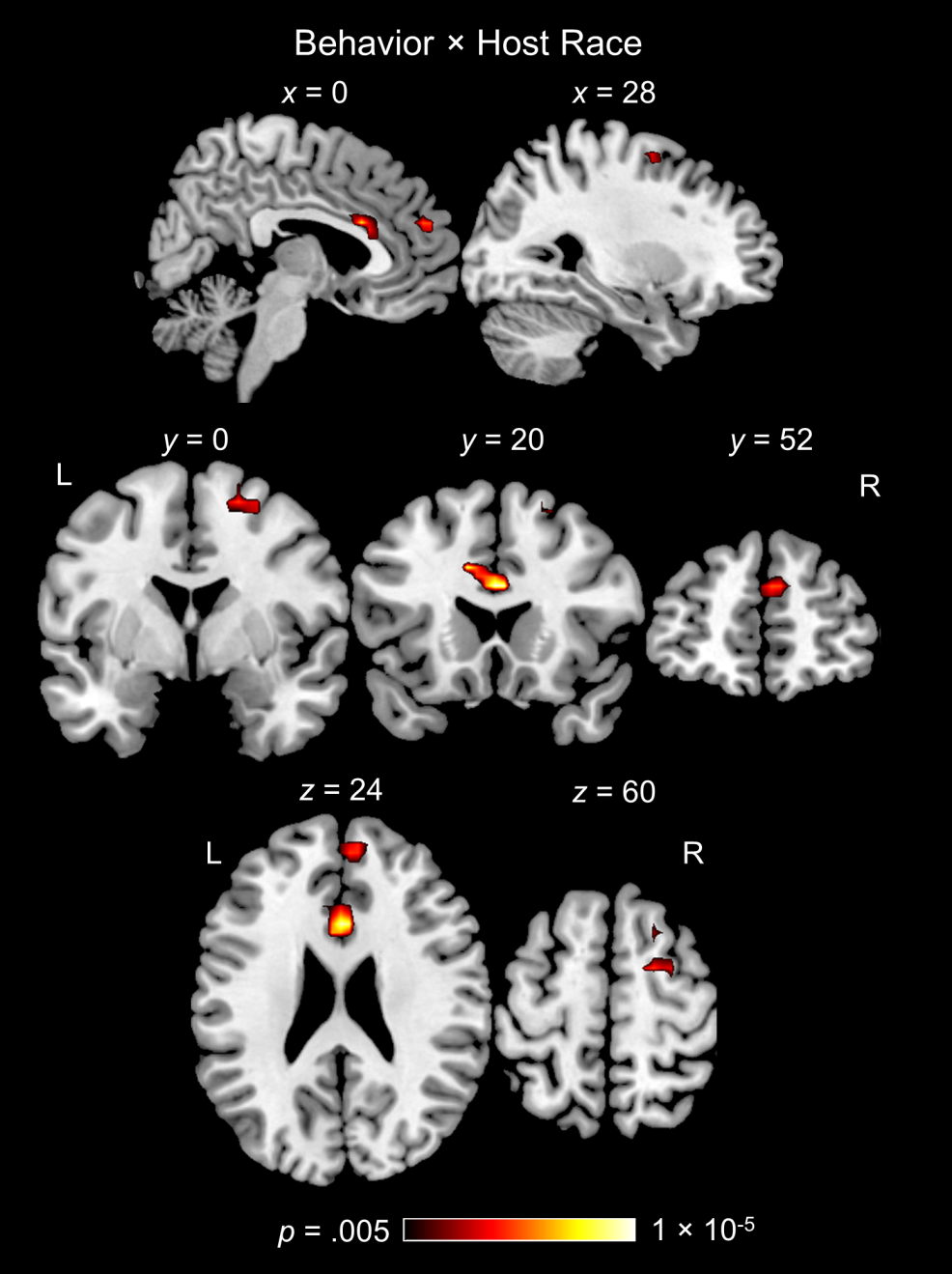
**

Figure S4 (A). Brain regions showing differential activations for observing ingroup vs. outgroup social encounters. This figure identifies brain regions showing a significant interaction effect between Behavior and Host Race within the a priori ROI mask.


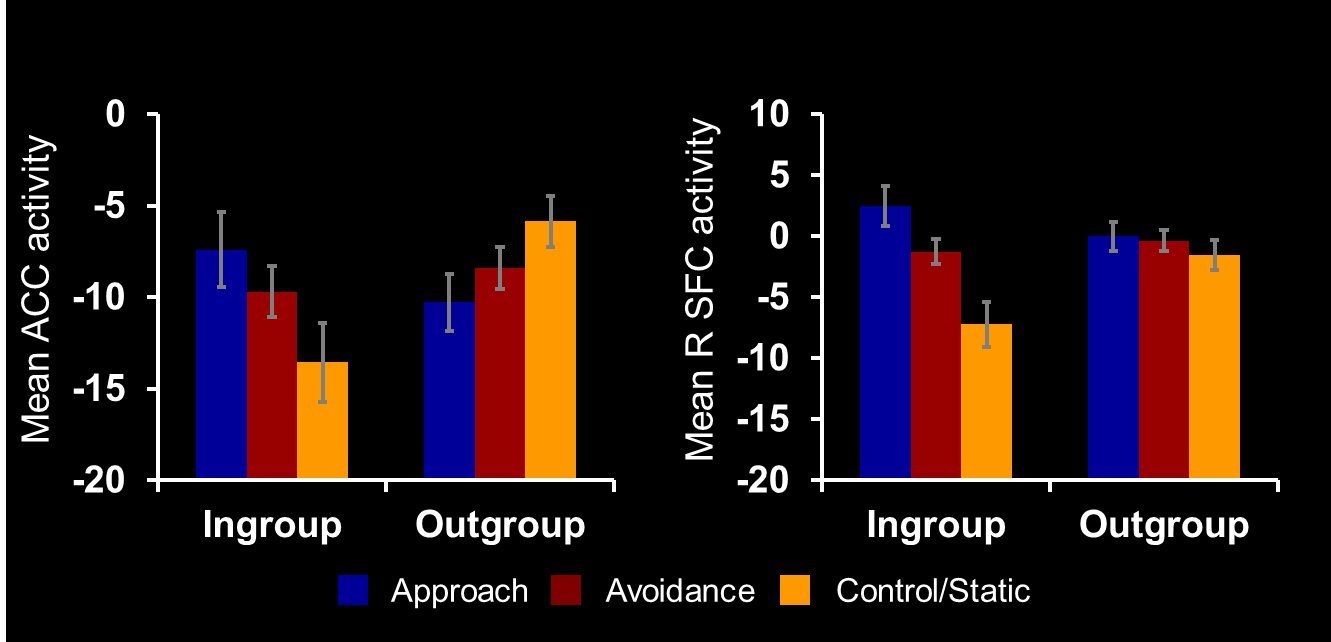


Figure S4 (B). Mean parameter estimates for the ACC and SFC clusters identified in a 3 (Behavior) × 2 (Host Race) ANOVA. The bar graphs indicate mean parameter estimates extracted from the ACC and right SFC clusters showing a significant interaction effect between Behavior and Host Race. Error bars indicate the standard error of the mean for each condition.

**S5. Modulation of functional connectivity linked to observing different types of behavior**

Following identification of the mPFC, ACC, and SFC regions showing differential activations for observing ingroup vs. outgroup social encounters, analyses of functional connectivity were performed as 3 (Behavior) × 2 (Host Race) ANOVAs using as seeds peak activity of these regions independently identified from the activation analyses. These ANOVAs identified a significant main effect of Behavior using as seeds the mPFC and the ACC. Post-hoc *t* tests were performed on the mean functional connectivity (i.e. *Z*-transformed *r* values) calculated within each significant cluster.

First, the mPFC showed increased connectivity with left pSTS/EBA (BA 18/19) and left middle frontal gyrus (BA 9) for observing dynamic social interactions than static social scenes (*t*[19] = 5.81, *p* < .001 and *t*[19] = 4.47, *p* < .001, respectively). Within the dynamic social interaction conditions, connectivity between the mPFC and these regions was also greater for observing avoidance than approach behaviors (*t*[19] = 2.22, *p* = .04 and *t*[19] = 2.38, *p* = .03, respectively), although the differences were smaller than those observed between the dynamic vs. static conditions.

Second, the ACC showed increased connectivity with bilateral pSTS extending into the surrounding parietal areas (e.g., inferior parietal lobule) (L: BA 39/40, R: BA 22/40) as well as the right middle/inferior frontal gyrus (BA 10/46) for observing avoidance behaviors than approach and static behaviors (R pSTS: *t*[19] = 4.35, *p* < .001 for avoidance vs. approach, and *t*[19] = 4.59, *p* < .001 for avoidance vs. static; similar differences were also observed for the left pSTS and the right frontal clusters). No significant differences in connectivity were identified between approach and static behaviors (all *t*’s < 1 and *p*’s > .05). Taken together, these findings suggest that the mPFC and ACC regions that are sensitive to different types of behaviors displayed by ingroup and outgroup members may also be involved in processing dynamic nonverbal behaviors at a more general level.

References

Dolcos, S., Sung, K., Argo, J. J., Flor-Henry, S., & Dolcos, F. (2012). The power of a handshake: Neural correlates of evaluative judgments in observed social interactions. *Journal of Cognitive Neuroscience, 24*(12), 2292-2305. doi: 10.1162/jocn_a_00295

Kubota, J. T., Banaji, M. R., & Phelps, E. A. (2012). The neuroscience of race. *Nature Neuroscience, 15*(7), 940-948. doi: 10.1038/nn.3136

Molenberghs, P. (2013). The neuroscience of in-group bias. *Neuroscience & Biobehavioral Reviews, 37*(8), 1530-1536. doi: 10.1016/j.neubiorev.2013.06.002

Morrison, S., Decety, J., & Molenberghs, P. (2012). The neuroscience of group membership. *Neuropsychologia, 50*(8), 2114-2120. doi: 10.1016/j.neuropsychologia.2012.05.014

Shkurko, A. V. (2013). Is social categorization based on relational ingroup/outgroup opposition? A meta-analysis. *Social Cognitive and Affective Neuroscience, 8*(8), 870-877. doi: 10.1093/scan/nss085

Tzourio-Mazoyer, N., Landeau, B., Papathanassiou, D., Crivello, F., Etard, O., Delcroix, N., Mazoyer, B., & Joliot, M. (2002). Automated anatomical labeling of activations in spm using a macroscopic anatomical parcellation of the mni mri single-subject brain. *NeuroImage, 15*(1), 273-289.
